# *Brd4* expression in CD4 T cells and in microglia promotes neuroinflammation in experimental autoimmune encephalomyelitis

**DOI:** 10.1186/s12974-025-03449-9

**Published:** 2025-06-02

**Authors:** Anup Dey, Matthew Butcher, Anne Gegonne, Ryoji Yagi, Keita Saeki, Eunju Lee, Dinah S. Singer, Jinfang Zhu, Keiko Ozato

**Affiliations:** 1 Division of Developmental Biology, NICHD, NIH, Bethesda, MD USA; 2 Laboratory of Immune System Biology NIAID, NIH, Bethesda, MD USA; 3 Experimental Immunology Branch, CCR, NCI, NIH, Bethesda, MD USA

**Keywords:** EAE, BRD4, MOG, CNS, Neuroinflammation, Brd4cKO, Microglia, Th17 T cells, RNA-seq

## Abstract

Microglia are resident innate immune cells in the central nervous system (CNS) that provides anti-microbial protection but also promote neuroinflammation. BRD4 is a chromatin reader that binds to acetylated histones and directs transcription of numerous genes. However, it is unknown whether and how BRD4 regulates microglia function. We addressed the role of microglia and BRD4 in a neuroinflammatory disease, experimental autoimmune encephalomyelitis (EAE), a mouse model for multiple sclerosis. It was reported earlier that in EAE, upon initial T cell activation in the peripheral lymphoid organs, CD4^+^ T cells migrate to CNS and are reactivated by resident or migratory antigen presenting cells resulting in full manifestation of EAE (Rossi and Constantin, Front Immunol 7:506, 2016), (Plastini et al., Front Cell Neurosci 14:269, 2020). Using conditional deletion of *Brd4* in CD4 T cells, we reveal that BRD4 regulates T helper cell differentiation and promotes T cell migration to CNS resulting in EAE. It remained unclear whether resident microglia are capable of reactivating migrating T cells to the CNS and if BRD4 plays a role in the process. To determine the role of microglial BRD4 in EAE, we constructed conditional knockout mice lacking *Brd4* (Brd4cKO) in microglia. RNA-seq analysis showed that *Brd4* deletion led to the downregulation of many microglia genes in both naive and EAE conditions. Consequently, Brd4cKO mice had markedly reduced EAE pathology, namely reduced paralysis, absence of axonal demyelination and inhibited expression of inflammatory cytokines. In vehicle treated mice (vehicle) abundant number of T cells were found to be near microglia that may lead to T cell- microglia interaction and T cell reactivation. In contrast, the number of T cells detected in the CNS of Brd4cKO mice was much fewer. This may lead to reduced T cell- microglia interaction, failure of T cells to get reactivated and hence failed to achieve full manifestation of EAE. These results demonstrate that microglia are critically involved in EAE disease progression for which BRD4 is essential. In summary, BRD4 directs transcription of genes defining microglia function. By so doing BRD4 promotes demyelination and neuroinflammation to exacerbate EAE.

**Supplementary Information:**

The online version contains supplementary material available at 10.1186/s12974-025-03449-9.

## Introduction

Multiple sclerosis (MS) is a neuroinflammatory disease of brain and spinal cord, caused by the infiltration of peripheral immune cells to the CNS from across impaired blood–brain barrier (BBB) resulting in widespread demyelination of myelin sheath and damage to the neuroaxonal fibers [1]. In experimental autoimmune encephalomyelitis (EAE), an animal model of MS, myelin specific CD4 autoreactive T cells are activated in peripheral lymph nodes, migrate to CNS and get reactivated by antigen presenting cells in the CNS [2]. Evidence shows that microglia, innate immune cells in CNS, play a critical role in modulating the brain parenchymal microenvironment. At steady state, microglia display a ramified morphology and engages in immune surveillance to help maintain CNS homeostasis. These functions have been ascribed to a distinct set of transcripts in the CNS [3, 4]. In the context of neuroinflammation, microglia proliferate and assumes morphological changes, changing from a ramified morphology to a shorter branched morphology. Following these morphological changes, microglia relocate to the site of injury or inflammation [5, 6]. In the EAE model, T cell entry to the CNS occurs together with the migration and accumulation of other lymphocytes, including macrophages, dendritic cells, and monocytes to the sites of demyelination. Microglia, other CNS associated macrophages, and invading myeloid cells all may contribute to the pathology of EAE [7, 8]. In some studies it has been postulated that invading monocyte derived macrophages play a central role in the immune response and in demyelination, while resident microglia have been proposed to be responsible for surveying damage, reducing inflammation and removing debris [9, 10]. More recently, however, interaction between microglia and regulatory T cells has been shown to be critical at the relapsing–remitting model for EAE [11]. Direct crosstalk between microglia and T cells have been suggested to be associated with neurodegeneration [12]. Furthermore, depletion of microglia by the colony stimulatory factor 1 receptor inhibitor, PLX5622 has been shown to diminish T cell proliferation, reactivation and delay in EAE onset [13].

The pathology of EAE depends on autoreactive CD4 + T cells, particularly Th17 and Th1/Th17 T cells that produce IL17 A, IFNγ, GMCSF and TNFα. BET bromodomain inhibitor has been shown to control Th17 differentiation by regulating Th17 cytokines IL17 A, IL21 and GMCSF and inhibited EAE symptoms in mice [14]. These results suggested that BET bromodomain proteins (BRD2, BRD3, BRD4 and BRDT) either collectively or individually, may contribute to T cell differentiation in the periphery following immunization with the antigen. Thus, studies with BET inhibitors have not fully clarified whether BRD4 is critical for eliciting EAE pathology. BRD4 is distinguished from other BET proteins for its C-terminal specific binding to ‘positive transcription elongation factor’ (pTEFb) that recruits additional coactivators resulting in RNA PolII phosphorylation and transcription elongation of many genes [15–17]. *Brd4* expression is mostly ubiquitous with abundant expression from early embryos to most adult tissues [18–21]. BRD4 is a cell cycle regulating protein that plays critical role in cell proliferation by regulating cell cycle specific genes [22]. In prior studies, we demonstrated that BRD4 is critically required for hematopoiesis from fetal liver and bone marrow and modulate inflammatory gene expression in peripheral macrophages [23]. Here, we constructed (i) CD4 + T cell and (ii) microglia-specific conditional Brd4cKO mice to determine the role of BRD4 in T helper cell activation in the peripheral lymph nodes and in microglia in the CNS, respectively. We observed that BRD4 is essential in T helper cell differentiation and proliferation in vitro (see Additional file 1) and mice with T cell specific Brd4cKO do not develop EAE symptoms due to defective T cell priming and effector differentiation. We show that mice with microglia specific Brd4cKO were similarly resistant to develop severe EAE symptoms. Brd4cKO microglia were defective in expressing genes required for the interaction with T cells and those involved in neuroinflammation and demyelination. Together, this study demonstrates a critical role of microglia and the requirement of BRD4 in eliciting EAE pathology.

## Materials and methods

### Mice

The generation of Brd4^f/f^ mice has been described previously by us [23]. Brd4^f/f^ mice were backcrossed to C57BL/6 background and were crossed to CD4^Cre^ (Jackson Laboratory strain #022071) and Cx3cr1^CreER^ (Jackson laboratory strain #020940, kindly provided to us by Yosuke Mukoyama). For induction of Cre- mediated recombination in microglia 6 to 8 weeks old Brd4^f/f^; Cx3cr1^CreER/+^ females, were injected intra-peritoneally with 2 mg Tamoxifen (Sigma), (20 mg/ml in corn oil), for 5 consecutive days. Littermates with similar genotype were injected with 100 µl corn oil and was used as vehicle treated controls. All experiments were approved by the National Institute of Child Health and Human development (Animal study Program #20–044, #23–044) and was carried out in accordance with National Institutes of Health guidelines.

#### EAE induction

Four weeks following Tamoxifen treatment, mice from both groups were immunized by subcutaneous injection with 200 µg of MOG_35–55_, containing1-5 mg heat killed Mycobacterium tuberculosis emulsified in Complete Freund’s adjuvant (EK-2110; Hooke Laboratories). Additionally, mice were injected with 100 ng of pertussis toxin intraperitoneally and were repeated once 24 h later. Mice were scored daily based on detectable EAE symptoms; tip of the tail is limp—0.5, limp tail—1.0, limp tail hind leg inhibition – 1.5, hind limb paralysis—2.0, front limb inhibition—2.5, paralysis on both front and hind limb—3.0.

### Immunohistochemistry

Animals were anesthetized using a combination of Ketamine 100 mg/kg and Xylazine 10 mg/kg body weight, respectively. Animals were perfused transcardially with phosphate buffered saline (PBS) followed by 4% paraformaldehyde (PFA). Spinal cords were fixed further with PFA overnight and subsequently transferred to 30% sucrose in PBS. For immunofluorescence study 30 µm cryosections were blocked and permeabilized with 3% goat serum and 1% triton-X for 1 h. For immunostaining sections were incubated overnight at 4^0^C with primary antibodies at a dilution of 1: 500 for rabbit anti-Iba1 (Fujifilm), 1:500 for rat anti-CD3 (Abcam clone; CD3-12), 1:200 for rat anti-IA/IE (Biolegend clone; M5/114.15.2). For detection of these primary antibodies, as appropriate, sections were incubated for 2 h at room temperature with Alexa fluor 488 and Alexa Fluor 633 conjugated goat secondary antibody (1:400, Invitrogen). Nuclei were detected by using Hoechst 33,342 (1 µg/ml, ThermoFisher). Images were acquired using a Zeiss LSM800 confocal microscope to determine number and morphology of microglia and number of invading T cells in the white mater. Cell number was evaluated using ImageJ software. Dendrite lengths and cell sizes were determined using Imaris software.

#### Histology

For histological analysis spinal cords were fixed with 4%PFA as described. Sections were embedded in paraffin before staining with Luxol fast blue and H&E to assess demyelination and neuronal invasion at the white mater. Histological images were obtained using a Zeiss Axiophot fluorescence microscope. The extent of demyelination was assessed using ImageJ software.

### Intracellular cytokine staining

Splenocytes, lymph node and CNS cells were isolated from immunized mice. For CNS mononuclear cell isolation, brain and spinal cord (CNS) homogenates were precipitated in 1X PBS. Cells were then purified using 70–30% neutral percoll gradient. Prior to staining aliquots of splenocytes and lymph node cells (1 × 10^6^) and neural mononuclear cells (5 × 10^4^) were restimulated using PMA (50 ng/ml) and ionomycin (1 µg/ml) with 1 µl/ml Monensin at 37^0^C for 6 h. After stimulation cells were washed with PBS and resuspended in 100 µl of staining buffer (1%BSA in PBS). To prevent nonspecific binding cells were incubated with Fc blocker (anti mouse CD16/CD32 antibody) on ice for 10 min, followed by incubation with various cell surface markers. CNS Mononuclear cells were split into two aliquots and were incubated either in T cell or in myeloid cell staining cocktail. For staining T cells, T cell specific antibody included FITC CD4 clone GK1.5; BV786 CD3 clone 17 A2; AF700 CD44 clone IMZ; AF405 Foxp3 Clone 1054 C. To identify microglia/macrophage cells, cocktail of antibody included Alexa Fluor 405 CD11b clone: M1/70; APC/Cy7 CD45 clone 30-F11; BV605 F4/80 clone BM8; Alexa Fluor 488 TMEM119 clone106-6, PE/Cy7 CD11c clone N41; Alexa Fluor 700 IA/IE clone M5/114; PE/Cy5 CD80 clone16-10 A1 in 100 µl of staining buffer on ice for 30 min. Cells were washed, followed by fixation and permeabilization using cytofix/cytoperm solution and stained for intracellular cytokines to detect IL17 A (PE/Cy7 IL17 A clone; TC11-18H10.1); IFNγ (BV605 IFNγ clone; XMG1.2); GMCSF (DsRed GMCSF) TNFα (PerCP TNFα clone:MP6-XT22). Cells were acquired using BD Fortessa and analyzed using FlowJo software, BD Bioscience.

#### RNA-Seq

For RNA-seq analysis 20–40,000 microglia cells were sorted directly in Trizol (Invitrogen), snaped freeze and stored at −80^0^C. RNA was isolated using Trizol-chloroform extraction. RNA-seq library was prepared using SmartSeq v4 Ultra Low input RNA Kit (Takara) and Illumina Nextera XT library preparation Kit. RNA-seq libraries were sequenced using NextSeq-500 system. 3 to 4 biological replicates were used in this study. Raw Fastq files were analyzed using NCBI pipeliner/4.0.2. For RNA-Seq analysis paired end reads were aligned to Mus musculus reference genome mm10, using splice-aware aligner STAR. DESeq2 software were used to determine differentially expressed genes with *P*-values and FDR. Transcript abundances were quantified using transcripts per million (TPM).

### Statistical analysis

Data are presented here as mean ± SD with number of repeats showed in figures. All statistical analyses were performed using GraphPad Prism 9.0. Comparison between two groups were analyzed using unpaired t test with Welch’s correction. Statistical significance of EAE clinical score was determined by Mann Whitney nonparametric test. Statistically significant was considered when *p*-value was < 0.05.

## Results

### *Brd4* expression in CD4 T cells is required for initial activation and subsequent infiltration of myelin specific effector T cells to CNS

**Fig. 1:**
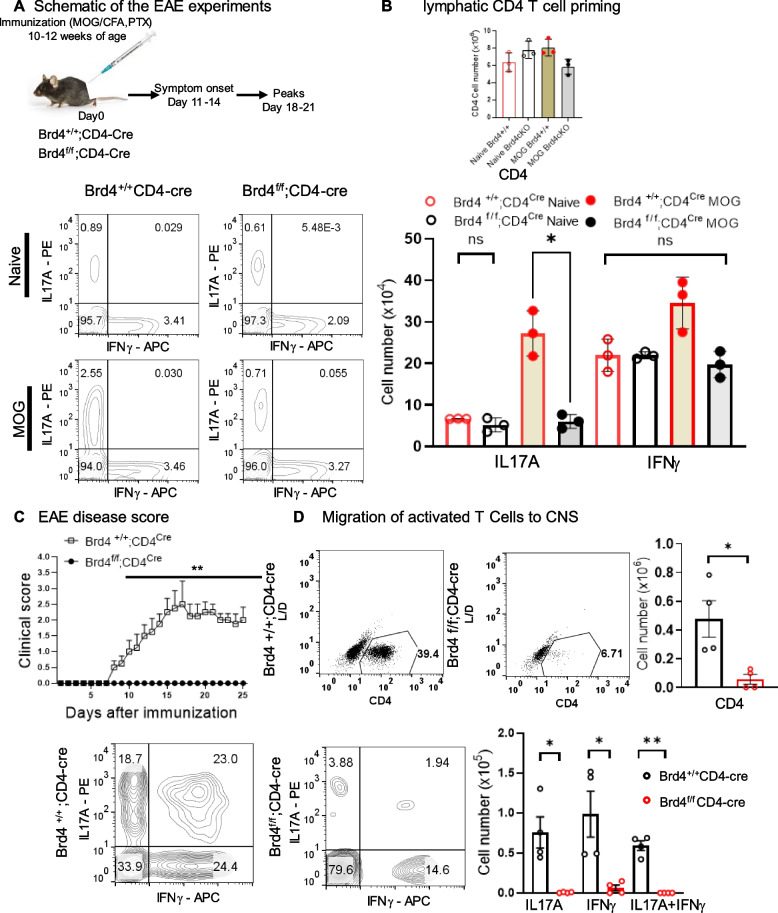
Brd4 deletion in CD4 T cells protects mice from EAE (**A**) Schematic showing EAE induction and its progression. Mice were immunized, and disease progression was monitored. **B** Cytokine expression analysis from lymph nodes of Brd4^+/+^; CD4^Cre/+^ and Brd4^f/f^; CD4^Cre/+^ mice in naïve and 5-day post immunization (3 mice were used per condition). Total number of T cells were similar in all conditions (top). T cell priming was evidenced by significantly higher IL17 A producing T cells in MOG treated Brd4^+/+^; CD4^Cre/+^ mice. While the number of IFNγ producing cells were similar in all conditions; flow cytometric analysis (below, left), quantification of cell number (below, right). **C** EAE progression among Brd4^+/+^; CD4^Cre/+^ (*n* = 4) mice, while EAE was prevented in Brd4^f/f^; CD4^Cre/+^ (Brd4cKO mice) (*n* = 4). Each data point is the average ± S.E.M., **p < 0.001 (**D**) (Top) Flow cytometric analysis of CNS invading CD4 T cells. Quantification of CD4 T cell number (right). (Bottom left) Flow cytometric analysis of corresponding T cells expressing IL17 A, IL17 A and IFNγ and IFNγ only, quantification on the right. Data presented as mean ± SD, **p < 0.01, *p < 0.05

By testing small molecule inhibitors, BET proteins have been identified to be critical for T cell activation [14, 24]. These inhibitors target bromodomains of all members of BET family proteins and function in a dose dependent manner. In the EAE model, it is well established that, in response to immunization with MOG antigen, naïve T cells differentiate to Th17 cells within draining lymph nodes and are critically involved in the pathogenesis of EAE once they migrate to the CNS [25]. Previous investigation showed that transient treatment of JQ1, a BET inhibitor that prevents chromatin occupancy of BET proteins, inhibits transcription of Th17 genes and ameliorates EAE severity [14]. To study the contribution of BRD4 excluding other BET proteins, we constructed Brd4cKO lacking *Brd4* in T cells (Brd4^f/f^CD4-Cre). We observed that naïve T cells from control mice (Brd4^+/+^CD4-Cre) supported T cell differentiation in vitro, but naïve T cells from Brd4cKO mice failed to support in vitro T cell differentiation (see Additional file 1).To generate EAE mice, 10 to 12 weeks old mice were immunized using an emulsion of myelin oligodendrocyte glycoprotein_35–55_ (MOG) peptide with complete Freund’s adjuvant and were co-injected with Pertussis toxin as described in the materials and methods section and in Fig. 1A legend. On day 5 following immunization, cytokine expression was monitored in CD4 T cells from draining lymph nodes. Total CD4 T cell numbers were similar among all conditions (Fig. 1B, upper panel) while IL17 A expressing cells were significantly lower in Brd4cKO mice relative to control mice (see Brd4^+/+^CD4 Cre—Brd4 control and Brd4^f/f^CD4 Cre—Brd4cKO, Fig. 1B, lower panels). No changes were observed between the genotypes when IFNγ expressing Th1 cells were quantified (Fig. 1B). Cells expressing both IL17 A and IFNγ were not detectable at this stage (Fig. 1B) [25]. Control mice developed EAE as expected with the onset of clinical symptoms on day 10 post immunization and reached the peak of EAE symptoms on day 18. Whereas Brd4cKO mice were resistant to EAE, and they did not develop EAE symptoms within the 25-day experimental period (Fig. 1C). Examination of cellularity from spinal cord and brain (hereafter CNS) revealed that in comparison to Brd4cKO, there was a massive infiltration of CD4 T cells in control (Fig. 1D). Consequently, higher number of CNS infiltrating CD4 T cells included those expressing IL17 A alone, IFNγ alone or highly proinflammatory T helper cells coexpressing IL17 A and IFNγ (Fig. 1D, lower panel). These data show that *Brd4* deletion in CD4 T cells prevented T cell activation and migration to the CNS thus preventing development of EAE. We conclude that BRD4 in T helper cells promotes EAE pathogenesis.

### *Brd4* deletion in microglia alone reduces EAE severity in mice

**Fig. 2:**
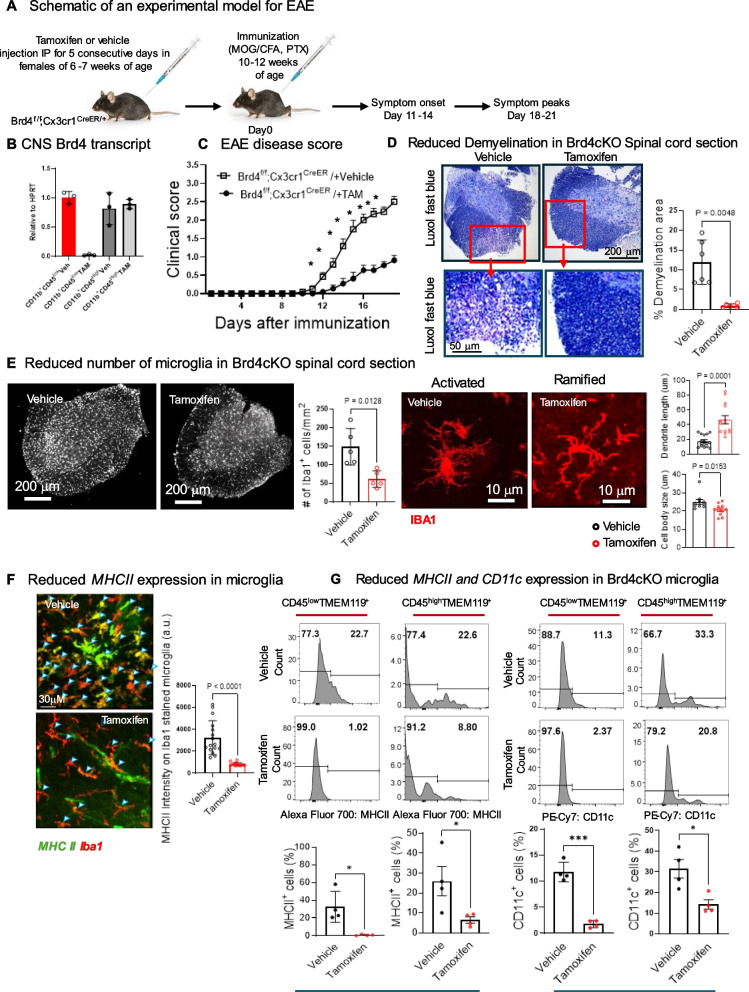
Effect of Brd4 deletion in microglia during EAE. **A** Schematic showing EAE induction and its progression. To delete *Brd4,* 6 to 7 weeks old Brd4^f/f^; Cx3cr1^Cre/+^ mice were treated with Tamoxifen for 5 consecutive days. 4 weeks post Tamoxifen or vehicle injection, mice were immunized, and disease progression was monitored. **B** qRT-PCR of *Brd4* transcript from microglia (CD45^low^CD11b^+^) and CNS invaded myeloid cells (CD45^High^CD11b^+^) isolated from vehicle and Tamoxifen treated mice respectively. Microglia and CNS invading myeloid cells were distinguished using surface markers CD45, CD11b, F4/80 and TMEM119 are presented in supplementary Fig. 1 A. **C** Following immunization EAE scores from Tamoxifen treated (Brd4cKO, reduced symptoms) and vehicle treated (EAE) mice were recorded. Data represents average ± SEM of Tamoxifen (*n* = 12) and vehicle (*n* = 12) treated mice from 3 independent experiments *p < 0.001. **D** Histology of spinal cord section using Luxol fast blue for assessing demyelination. Scale bars, 200µm (top), 50µm (below) showing intense demyelinated region on white matter in vehicle treated mice. Quantification of demyelination (right), data is average ± S.D. from two independent experiments. **E** Immunohistochemistry of spinal cord section stained with Iba1 assessed microglia population (left). Quantification using ImageJ shows twofold increase in number (middle). Reactive morphology among vehicle treated samples, while ramified morphology among Tamoxifen samples (right). Dendrite length and cell body size measured using Imaris software (far right) (**F**) Immunohistochemistry of spinal cord section showing colocalization of Iba1 stained microglia and MHCII. Intensity of MHCII was measured using ImageJ. MHCII staining was stronger in vehicle. (Right) Strong MHCII expression is in vehicle (black symbol), while Brd4cKO had weaker MHCII expression (Tamoxifen) (red symbol). **G** Flow cytometry plots (top) and corresponding quantification (bottom) of MHCII and CD11c expression from CD45^low/high^CD11b^+^TMEM119^+^ microglia. MHCII and CD11c expression were stronger in vehicle on the left (black symbol) while were weaker in Brd4cKO (Tamoxifen) on the right (red symbol) Data presented as mean ± SD, ***p < 0.001, *p < 0.05

Naïve autoreactive T cells are activated during EAE by myelin oligodendrocyte glycoprotein_35–55_ (MOG) in draining lymph nodes triggering lymphoid cells and other myeloid cells to invade into the CNS through damaged BBB. In our EAE model, BBB integrity likely was broken in both vehicle and Brd4cKO [26], confirmed by the intense Evan’s blue extravasation (see Additional file 2) and equal influx of myeloid cells (Figure S1D). Upon entry to CNS, T cells come in contact with a milieu of antigen presenting cells and are activated further [27]. While evidence indicates that microglia to contribute to EAE, this issue has remained elusive. Thus, to ask whether Brd4 in microglia affects EAE pathology: we constructed Brd4cKO mice using Brd4^f/f^ Cx3cr1^CreER^ strain. *Brd4* was deleted upon tamoxifen injection (Fig. 2A) [7]. Vehicle (corn oil) injected Brd4^f/f^ Cx3cr1^CreER^ mice were used as control (vehicle). Microglia were identified by FACS analysis from spinal cord and brain (hereafter CNS) (Figure S1 A). Myeloid cells were sorted as CD45^low^F4/80^+^TMEM119^+^ (microglia) and CD45^high^F4/80^+^TMEM119^−^ (invading myeloid cells). Efficient *Brd4* deletion was confirmed by qRT-PCR, where in Brd4cKO, CD45^low^ population (microglia), *Brd4* transcript was not detectable, while robust *Brd4* transcript was detected in vehicle (CD45^low^) and in CD45^high^ populations (Fig. 2B). EAE clinical score for disease progression was monitored daily for 18 to 20 days. Graph in Fig. 2C is an average of 3 independent sets of experiments, with 4 mice per group in each set. Clinical symptoms appeared in vehicle treated mice around day 12 and peaked by day 16–18. Brd4cKO mice exhibited milder symptoms during the period studied. This observation indicated that BRD4 function in microglia drives EAE pathogenesis. Tamoxifen effect on EAE symptoms was tested by treating Brd4^f/f^ mice with tamoxifen. After immunization these mice displayed similar course of pathogenesis as vehicle treated mice (see Additional file 3).

Demyelination in white matter is one of the salient features of EAE pathogenesis. We stained spinal cord sections from vehicle and Brd4cKO mice with Luxol fast blue to determine the extent of myelin damage on white matter. Widespread demyelination was noticed throughout the length of spinal cord sections from vehicle treated mice, while myelin layer was intact among Brd4cKO and nonimmunized spinal cord (Fig. 2D, Additional file 4). Shown on the right is the extent of demyelination, three data points each were taken from two mice. Noticeably, myelin damage in the vehicle accompanied significantly higher level of invading cells shown by the H & E stain, reduced cell migration was seen in Brd4cKO and nonimmunized spinal cord (Figure S1B, Additional file 4). Immunohistochemistry using Iba1 antibody detected a 2–fivefold increase in the number of microglia in the spinal cord of vehicle treated mice relative to Brd4cKO mice (Fig. 2E). This may be due to defective microglia proliferation in the early stages of EAE, given that BRD4 is known to promote proliferation [28, 29] [22]. Morphologically, microglia from Brd4cKO were highly ramified indicative of homeostatic surveillant type, while microglia from vehicle treated mice with EAE phenotype had numerous and shorter dendrites, with larger cell body often seen in stress conditions (Fig. 2E, right panel) [30].

We addressed *MHCII* expression using immunohistochemistry and flow cytometry. Strong *MHCII* expression colocalizing I*ba1* staining were detected on vehicle treated microglia. *MHCII* expression was found to be much weaker in *Brd4* deleted microglia (Fig. 2F). This observation was confirmed further by flow cytometry, where once again reduced *MHCII* expression was noted in Brd4cKO microglia (Fig. 2G, see CD45^low/high^ TMEM119^+^ cells). *CD11c* expression in microglia have been linked to aging and disease condition including MS and EAE [31–33]. We observed reduced *CD11c* expression in *Brd4*cKO microglia (Fig. 2G). Additionally, reduced expression of *CD80* costimulatory molecules was observed in *Brd4* deleted microglia (Figure S1 C). It is notable that with the breakdown of BBB integrity peripheral myeloid cells accessed to CNS unobstructed and expressed abundant *MHCII, CD11c* and *CD80* in both vehicle and Brd4cKO (Figure S1D). These results indicated that BRD4 in microglia, not infiltrating myeloid cells, played a direct role in EAE development.

### Brd4 deletion in homeostatic microglia

**Fig. 3:**
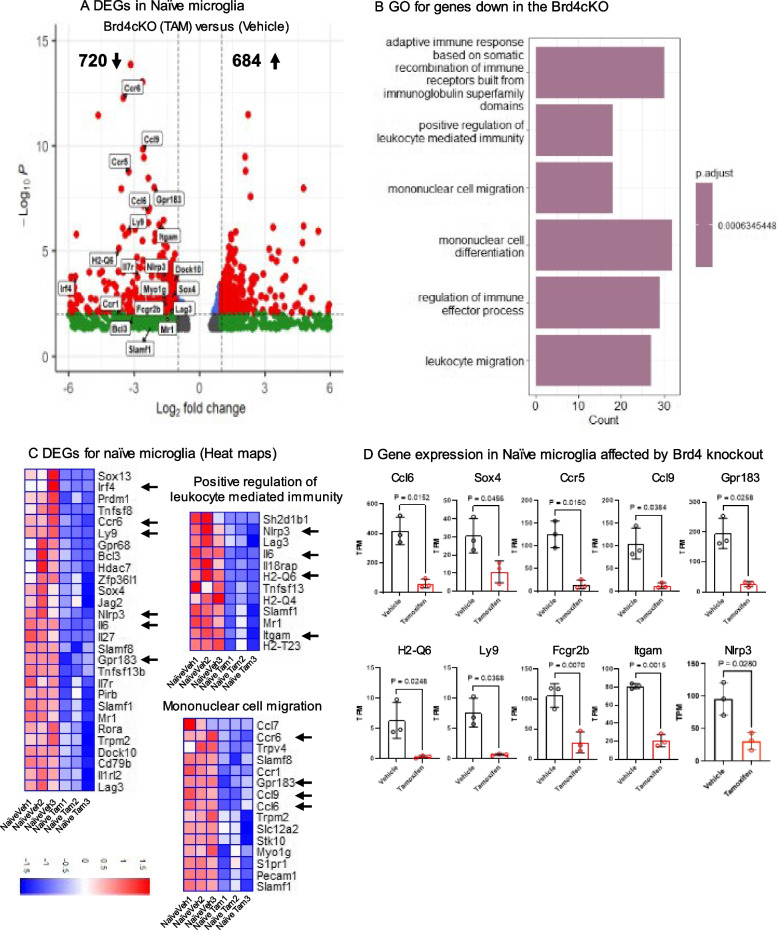
Transcriptome profiling of deleted naïve microglia (**A**) Volcano plot comparing naïve microglia from Brd4cKO (Tamoxifen) and vehicle treated mice. Significant changes in expression (fold change > 2, *p* value < 0.05) are shown in red dots. **B** GO analysis of 720 downregulated genes in Brd4cKO (Tamoxifen) relative to ‘vehicle’ microglia using clusterProfiler package in R. **C** Heat maps of genes from selected GO categories. **D** Quantification of relative TPM values of representative DEG’s selected from heat maps on C (arrows). Significance of differences were determined by unpaired t-test with Welch’s correction

BRD4 is critically important in controlling differentiation and function of many immune cells, as it regulates transcription in a cell type specific manner [23, 34, 35]. Given that *Brd4* deletion in microglia caused profound impact on EAE pathology in mice, studying BRD4 regulated gene expression was of high interest. We performed whole genome transcriptome analyses for naïve microglia from vehicle and Brd4cKO (Brd4^f/f^;Cx3cr1^CreER^) mice [7]. CNS mononuclear cells were isolated 4 weeks after Tamoxifen treatment, microglia were identified as CD11b^+^CD45^low^F4/80^+^TMEM119^+^ and isolated by FACS sorting (Figure S2 A). Purified single cell suspension was subjected to RNA-seq analysis. We used DESeq2 and used fold change > 2 and *P* < 0.05 threshold to assess the difference in the gene expression between vehicle and Brd4cKO naïve microglia. With these criteria 720 genes were down regulated while 684 genes were upregulated in naïve Brd4cKO microglia (Fig. 3A). Gene ontology analysis using clusterProfiler software revealed down regulation of transcripts of various categories (Fig. 3B) that includes genes related to mononuclear cell differentiation (*Dock10, Sox4, Bcl3, Il7r *etc*.*), mononuclear cell migration (Ccl7*, Ccl6, Ccr1, Ccl9, Ccl6, Trm2, Myo1 g, Slamf1*) and positive regulation of leukocyte mediated immunity *(Nlrp3, Lag3, Il6, MR1)* (Fig. 3C, D). These data indicated that BRD4 is required for establishing microglia response to external stimuli, cell migration and mediating immunity. However, it is important to note that transcription of microglia homeostatic genes was unaltered between vehicle and Brd4cKO (Figure S2B). A substantial number of genes were upregulated in Brd4cKO microglia (684). GO analysis using clusterProfiler software identified these genes to: ATP metabolic process, generation of precursor metabolites and energy etc. (see Additional file 5).

### Brd4cKO microglia does not express genes induced after EAE

**Fig. 4:**
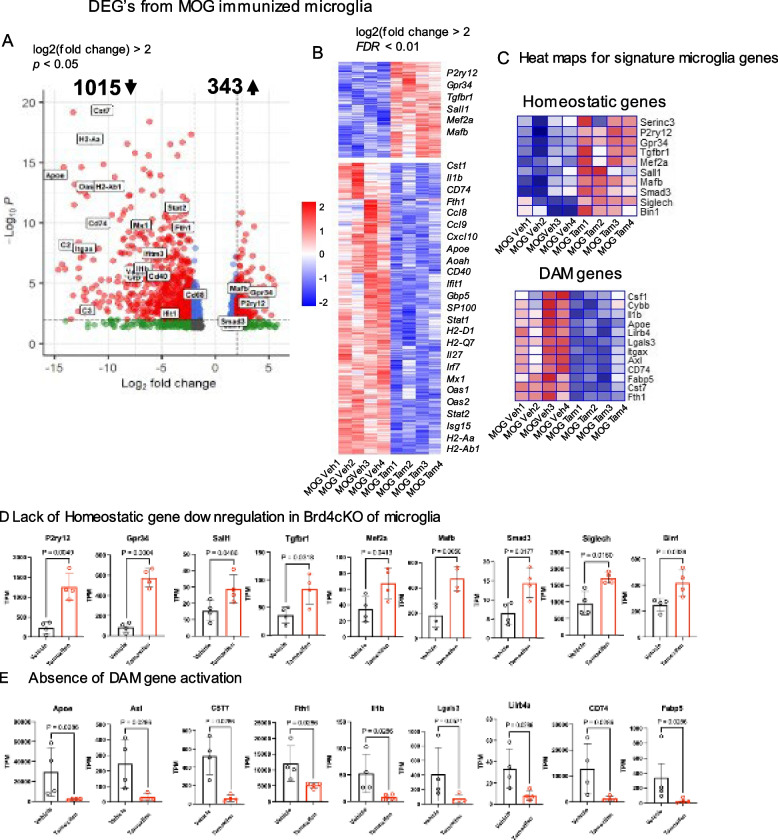
Unique transcriptional profile of Brd4cKO microglia during EAE (**A**) Volcano plot compares microglia gene expression from Brd4cKO (Tamoxifen) versus vehicle treated mice. Differential gene expression was determined using DESeq2 package, significant changes in expression log2 (fold change) > 2, *p* value < 0.05) are shown in red dots. **B** Heat map derived from 867 differentially expressed genes that were significantly altered in Brd4cKO microglia log2 (fold change) > 2, FDR < 0.01. **C** Hierarchical clustering distinguishing microglia identifying genes in homeostatic, and disease associated conditions. **D** Quantification of relative TPM values of representative DEG’s selected from heat maps on C. Significance of differences were determined by unpaired t-test with Welch’s correction

We next performed RNA-seq analysis of vehicle and Brd4cKO microglia from EAE mice (18 days post MOG treatment) and estimated DEGs using the same criterion as above. We found 343 genes were up regulated and 1015 genes were down regulated in Brd4cKO microglia (Fig. 4A). Among down genes 177 were common to both naïve and MOG treated microglia (Figure S2 C), but their relative expression was higher in MOG treated microglia. Metascape software analysis revealed that these common down regulated genes are important for leucocyte activation, migration and immune response functions (Figure S2D). Microglia from immunized vehicle CNS, we found reduced expression of signature homeostatic markers such as, *P2ry12, Siglech, Gpr34, Hexb, Sall1, Tgfbr1, Mef2a, Mafb, Bin1, Smad3, *[6] (Fig. 4B, C, D), while they expressed at a normal level in Brd4cKO microglia. Concurrent with the loss of homeostatic microglia markers, there was a sharp increase in the expression of disease associated microglia genes (DAM), such as *Csf1, Il1b, Apoe, Cd74, Cst7, Axl, Lgals3* and *Fth1* in vehicle, but not in *Brd4*cKO microglia (Fig. 4B, C, D).

**Fig. 5:**
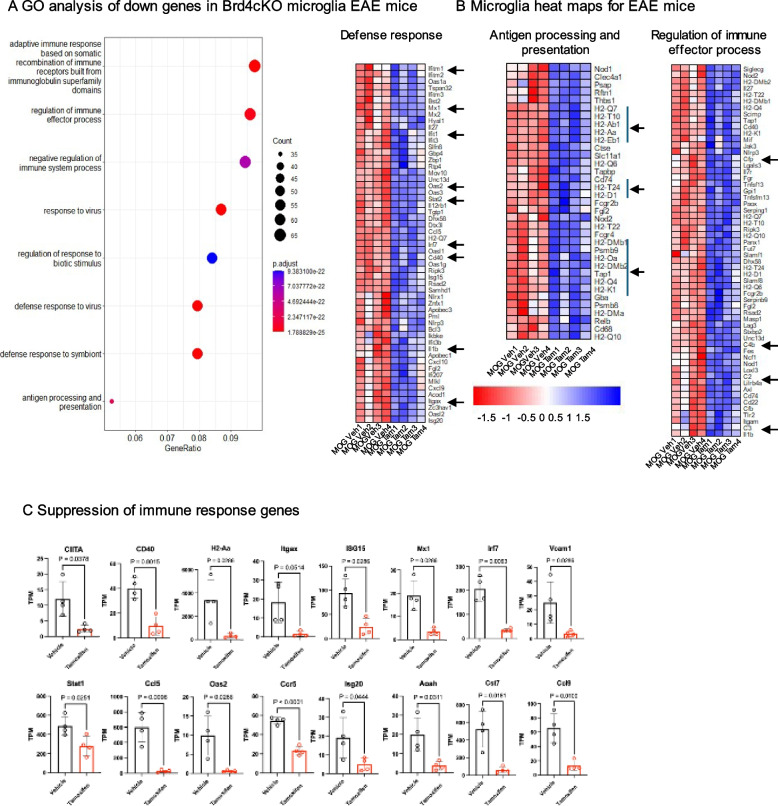
Contrasting microglial transcriptome from vehicle and Brd4cKO Brd4^f/f^; Cx3cr1^Cre/+^ mice at the peak EAE. **A** GO analysis of 1015 genes down regulated in Brd4cKO microglia (from Fig. 4), using clusterProfiler package. **B** Heat maps derived from genes in GO categories, such as defense response, antigen processing and presentation and regulation of immune effector process. **C** Quantification of relative TPM values of representative immune response genes from ‘vehicle’ and Brd4cKO mice. Significance of differences were determined by unpaired t-test with Welch’s correction

Gene ontology enrichment analysis further revealed that down regulated transcripts in Brd4cKO were related to defense response, regulation of immune effector processes as well as antigen processing and presentation (Fig. 5A). Function of microglia as an antigen presenting cell has been discussed in the past [36, 37]. In EAE for efficient adaptive immune response of T cells in the CNS, after its entry to the CNS, T cells would require additional antigen presentation, cytokine signal from antigen presenting cells and costimulatory activity. Given that deletion of Brd4 from microglia rescued mice from severe disease, it may be possible but not proven that Brd4cKO microglia are defective in antigen presentation.

Consistent with this view *MhcII, Cd74, Ciita, Itgax (Cd11c)* were all down regulated in Brd4cKO microglia. In addition, various chemokines such as, *Ccl5, Cxcl9 and Cxcl10,* genes in the complement system such as, *C2, C3, C4b* were downregulated. Likewise, interferon stimulated genes such as, *Mx1, Ifit1, Irf7, Oas2, IfitM1, Gbp6 and Stat1* were downregulated *in Brd4cKO microglia* (Fig. 5B and C). Various T cell costimulatory molecules expressed in microglia such as *CD40, CD80 and CD86* have been identified for T cell-microglia interaction. Upregulation of CD40 on microglia has been linked to T cell proliferation and infiltration of other leukocytes during EAE progression [28]. It was reported that in EAE and MS, Th cells express *CD40L* [27, 38]. We found that *Cd40* gene expression was downregulated in Brd4cKO microglia, suggesting disruption in T cells-microglia interaction (Fig. 5B, C). In other studies, with MS and EAE it was demonstrated that interaction of activated T cells and microglia via VCAM1 was important for TNF production from microglia causing inflammation, infiltration of peripheral immune cells and demyelination [27, 39]. We found that Vcam1 expression was down regulated in Brd4cKO microglia (Fig. 5C). Aggravated demyelination was reported in cuprizone mediated MS using Cst7 knockout mice [5, 40]. Cst7 was significantly downregulated in Brd4cKO microglia, suggesting reduced demyelination in Brd4cKO mice (Fig. 5C).

### CNS with Brd4cKO microglia had fewer T cells

**Fig. 6:**
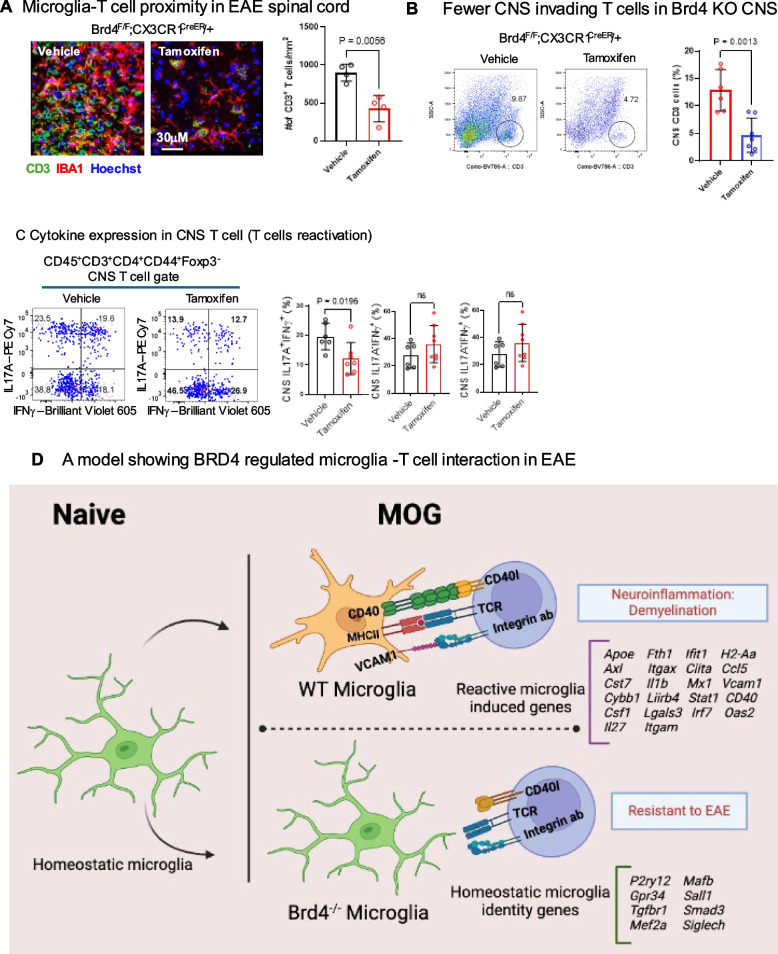
CNS infiltration of CD3 T cells from ‘vehicle’ and ‘Tamoxifen’ treated, Brd4^f/f^; Cx3cr1^Cre/+^ mice. **A** Immunohistochemistry of spinal cord section showing localization of Iba1 stained microglia and anti-CD3 labelled T cells (left). CD3 cell count using ImageJ software shows increased number of T cells in ‘vehicle’ (right). Significance of differences were determined by unpaired t-test with Welch’s correction. **B** Flow cytometry analysis showing reduced CD3 T cells in Brd4cKO CNS. Quantification of CD3 T cells (right), data was obtained from 6 WT and 7 Brd4cKO mice average ± S.D. Significance of differences were determined by unpaired t-test with Welch’s correction. **C** Flow cytometry and quantification of CNS Th17 A and IFNg cytokine expression (*n* > 6). **D** Model for BRD4 control of microglia-T cell interaction in EAE CNS. In WT microglia, expression of Vcam1, MhcII, Cd40 promote interaction via integrin, TCR and CD40 l on T cells. This leads to T cell proliferation and reactivation, microglia activation and upregulation of microglial proinflammatory and disease associated genes resulting in demyelination and axonal loss. Conversely, in Brd4cKO microglia, homeostatic genes were not downregulated, DAM genes were not induced, but genes necessary for the interaction with T cells, neuroinflammation and demyelination are severely downregulated, resulting in reduced EAE pathology

Our findings that genes critical for microglia- T cells interaction was downregulated in Brd4cKO microglia, prompted us to examine T cell number in the EAE CNS. First, we checked whether Brd4 deletion in microglia altered T cell in the periphery by testing T cells from spleen. FACS analysis revealed that Brd4 deletion in microglia did not affect the number of T cells in spleen, nor did the expression of *IL17 A, IFNγ, GMCSF and TNFα* changed (Figure S2E). This observation indicated that depletion of Brd4 in microglia did not alter activated T cell population in periphery. Next, we examined T cell population in CNS. For detecting invading T cells, spinal cord sections were stained with CD3 antibody. In vehicle treated samples we detected accumulation of CD3 positive T cells, surrounded by *Iba1* positive microglia. In contrast only a few CD3 positive T cells were detected in the CNS of Brd4cKO spinal cord (Fig. 6A). The number of T cells shown on the right was an average of 2 sections from 4 mice corroborated reduction of CNS T cells. Reduction of T cell population in CNS was confirmed further by flow cytometry (Fig. 6B). Additional FACS analysis in Fig. 6C showed that the percentage of IL17 A/IFNγ producing cells was lower in Brd4cKO CNS. Reduced T cell number in Brd4cKO condition together with the reduction of co-stimulatory molecules, CD40 and CD80 may lead to reduced T cell-microglia interaction causing the inhibition of EAE phenotype. Similar observation of reduced T cell population in CNS was reported earlier where PLX5622 was treated to deplete microglia and meningeal macrophages in EAE context [13].

A diagram in Fig. 6D depicts a model in which WT microglia interact with invading T cells, present MOG antigens, reactivate T cells and exacerbate EAE pathology. Whereas Brd4cKO microglia with reduced interaction with incoming T cells, reduces reactivation, decreasing EAE pathology. (see Discussion for details).

## Discussion

In this study we investigated the function of BRD4 in Th1/Th17 effector T cells and microglia in the CNS during EAE. We first show that mice with Brd4cKO T cells do not develop EAE, indicating that BRD4 in T cells promotes EAE. Our results extend the previous report that BET inhibitors reverse EAE pathology and illustrate the importance of BRD4 among other BET proteins [14, 41].

Nevertheless, the central focus of this study was to explore the role of BRD4 in microglia in the context of EAE. We felt it is important to first clarify the contribution of microglia in EAE, since this issue has remained somewhat inconclusive. Namely, the role of microglia has been debated in the context of MHCII, as results could be attributed to infiltrated myeloid cells [11, 37]. We show that mice with Brd4cKO microglia, which carry unaltered peripheral myeloid cells, do not develop full EAE, rather show reduced demyelination, less neuroinflammation and reduced paralysis. Our data provide convincing evidence that microglia contribute to EAE progression. These results are in line with the report that microglia ablation by PLX5622 inhibitor results in delayed onset and reduced EAE phenotype [13].

Further, we show that BRD4 is a major factor that confers the functional activity of normal microglia. In addition, BRD4 helps drive EAE progression. Little, if any information has been available to the role of BRD4 in microglia so far. The present study provides convincing evidence that BRD4 is required for microglia function and transcriptome programs in health and disease. Besides microglia, BRD4 plays a critical role in gene expression and function of various other cell types. For example, BRD4 is required for the development of hematopoietic stem cells and modulates inflammatory responses in peripheral macrophages [23, 42].

One of the most significant findings in this study was that Brd4cKO microglia did not interact with invading T cells in the CNS. Earlier reports showed that in MS and EAE microglia and T cells were in proximity near the lesion, they interacted via cytokines and chemokines [11, 27] and the extent of damage was correlated by the number of MHCII carrying microglia and number of T cells [43, 44]. Microglia ablation using PLX5622 inhibitor revealed a sharp decline in the number of infiltrated T cells, due to the decrease in T cell proliferation in the spinal cord resulting in delay in EAE onset [13, 45]. Using immunohistochemistry and flow cytometry we detected significantly fewer T cells in the CNS with Brd4cKO microglia. This may lead to reduced T cell- microglia interaction in the CNS. Transcriptome analysis provided mechanisms by which BRD4 controls microglia function under normal and EAE conditions. Brd4cKO microglia did not express DAM genes that were expressed in vehicle treated microglia in EAE, nor did they downregulate homeostatic genes, indicating that BRD4 provides transcriptome programs specific for EAE. It has been shown that T cells infiltrate brain parenchyma via vascular cell adhesion protein (VCAM1) expressed on microglia [43, 46]. The reduction of invading T cells into the CNS observed with Brd4cKO microglia is likely explained at least in part by downregulation of *Vcam1* expression.

Transcriptome data revealed that genes involved in antigen processing and presentation are downregulated in Brd4cKO microglia, including chemokines, such as *Cxcl10, Ccl5*, antigen presentation such as, *Ciita, Cd74 and H2-2a.* Thus, Brd4cKO microglia may possibly be defective in presenting MOG antigens to incoming T cells, therefore were unable to reactivate them. Furthermore, among other genes in the antigen presentation repertoire microglia expresses CD40, a costimulatory molecule that directly interact to T cells via CD40 ligand on T cells. It has been reported that this interaction is important for T cell expansion and continued infiltration of leukocytes to promote EAE disease progression [28, 38]. As *CD40* expression was reduced in Brd4cKO microglia, it would further undermine T cell-microglia interaction, resulting in inhibition of T cell expansion. Clinical symptoms in EAE accompanies demyelination of the CNS. Demyelination was reduced in Brd4cKO mice. Significantly, we found demyelination related genes such as *Cst7, Ccl6* and remyelination related genes such as *MHC II* and *Cd74* were downregulated in *Brd4* depleted microglia [5]. Further, inflammatory genes such as *Il1b, Ifit1, Mx1* were also downregulated in Brd4cKO microglia, likely contributed reduced neuroinflammation (Fig. 6D for a model). In summary, our data reveal that BRD4 in microglia promotes EAE by directing multiple gene sets that collectively drive EAE progression.

## Conclusion

In EAE Brd4 deletion in microglia alleviated neuroinflammation and suppressed EAE. Our data suggest that T cells entry to the CNS prompted activation of BRD4 regulated expression of inflammatory and activation related genes, possibly enhancing antigen presentation, T cell interaction, axonal demyelination and disease progression. Brd4 deletion inhibited inflammatory gene expression, resulting in fewer T cells and suppression of EAE.

## Supplementary Information

Supplementary Material 1: Supplemental Figure 1.

Supplementary Material 2: Supplemental Figure 2.

Supplementary Material 3: Table S1.

Additional file 1.

Additional file 2.

Additional file 3.

Additional file 4.

Additional file 5.

## Data Availability

RNA-seq data are available at https://www.ncbi.nlm.nih.gov/geo/query/acc.cgiaccessionnumberGSE275400. under accession number GSE275400.
