# Supplementary material for: Brd4 expression in CD4 T cells and in microglia promotes neuroinflammation in experimental autoimmune encephalomyelitis
Source: J Neuroinflammation. 2025 Jun 2;22:148. doi: 10.1186/s12974-025-03449-9 (PMC12131476; doi:10.1186/s12974-025-03449-9)
Supplement: Supplementary file 1 — Supplementary Material 1: Supplemental Figure 1. [file 12974_2025_3449_MOESM1_ESM.pdf]

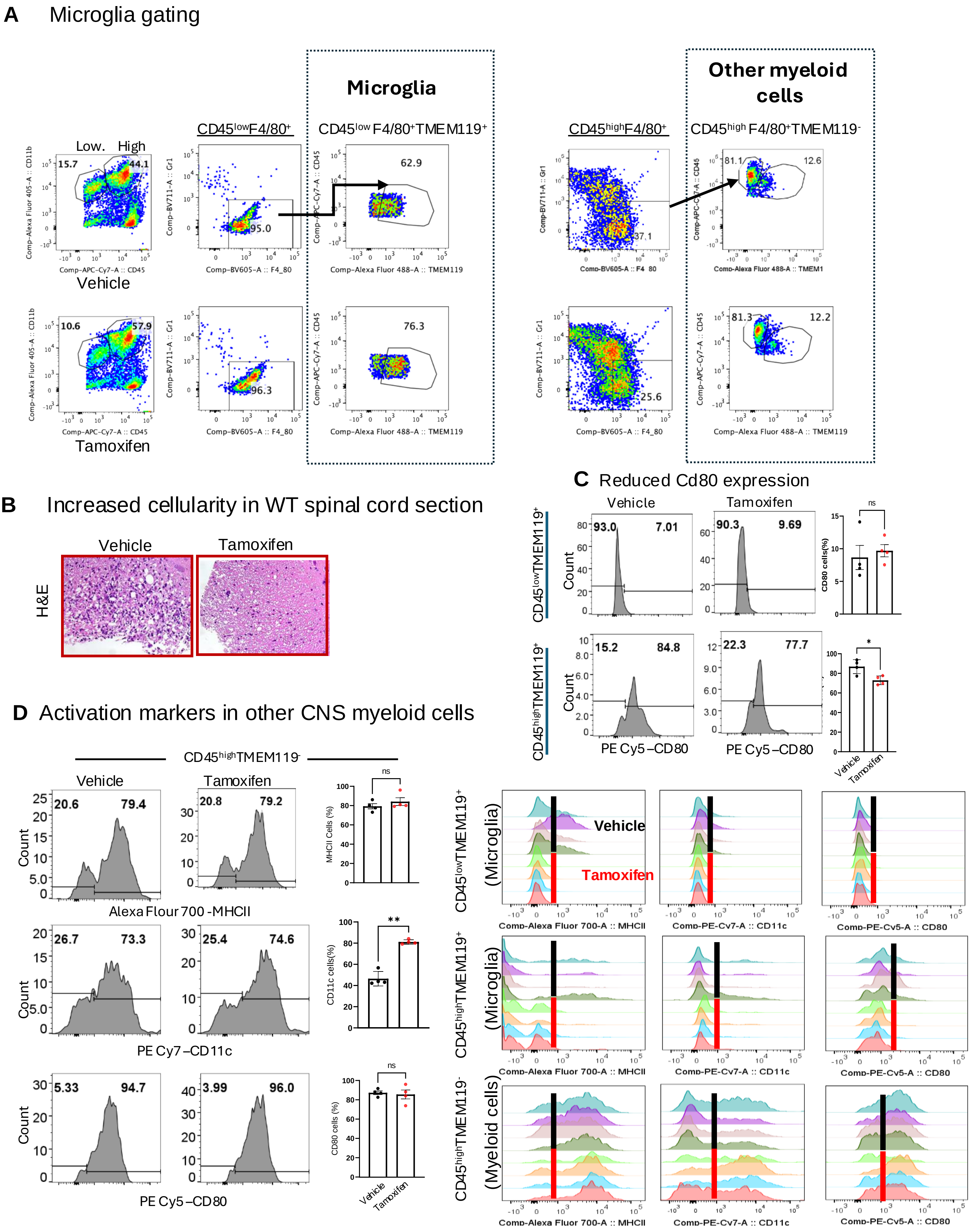
A Microglia gating
Microglia
Other myeloid cells
CD45lowF4/80+
CD45low F4/80+TMEM119+
CD45highF4/80+
CD45high F4/80+TMEM119-
Low. High
Vehicle
Tamoxifen
Comp-APC-Cy7-A :: CD45
Comp-BV605-A :: F4_80
Comp-Alexa Fluor 488-A :: TMEM119
Comp-Alexa Fluor 405-A :: CD11b
Comp-BV711-A :: Gr1
15.7
44.1
95.0
62.9
37.1
81.1
12.6
10.6
57.9
96.3
76.3
25.6
81.3
12.2
B Increased cellularity in WT spinal cord section
H&E
C Reduced Cd80 expression
CD45lowTMEM119+
CD45highTMEM119+
Count
93.0
7.01
90.3
9.69
15.2
84.8
22.3
77.7
PE Cy5 –CD80
CD80 cells(%)
ns
*
D Activation markers in other CNS myeloid cells
CD45highTMEM119-
20.6
79.4
20.8
79.2
Alexa Flour 700 -MHCII
MHCII Cells (%)
26.7
73.3
25.4
74.6
PE Cy7 –CD11c
CD11c cells(%)
**
5.33
94.7
3.99
96.0
CD80 cells (%)
CD45lowTMEM119+ (Microglia)
CD45highTMEM119+ (Microglia)
CD45highTMEM119- (Myeloid cells)
Comp-Alexa Fluor 700-A :: MHCII
Comp-PE-Cy7-A :: CD11c
Comp-PE-Cy5-A :: CD80

Supplemental Figure 1 Gating strategy for ex-vivo analysis of microglia. (A) representative strategy for flow cytometry analysis and microglia sorting for RNA-seq. (B) Histological section of spinal cord stained with H&E. Influx of invading cells were detected in WT section shown as black dots, reduced in Brd4cKO section. (C) Flow cytometry plots and quantification of CD80 co stimulatory molecule showing elevated CD80 expression in $CD45^{high}CD11b^{+}TMEM119^{+}$ WT microglia but not in Brd4cKO microglia. (D) (Left) Flow cytometry quantification of MHCII, CD11c, CD80 from $CD45^{high}CD11b^{+}TMEM119^{-}$ myeloid cells. (Right) Flow cytometry quantification of MHCII, CD11c, CD80 from $CD45^{low/high}CD11b^{+}TMEM119^{+}$ microglia and $CD45^{high}CD11b^{+}TMEM119^{-}$ myeloid cells from CNS.
