# Supplementary material for: Brd4 expression in CD4 T cells and in microglia promotes neuroinflammation in experimental autoimmune encephalomyelitis
Source: J Neuroinflammation. 2025 Jun 2;22:148. doi: 10.1186/s12974-025-03449-9 (PMC12131476; doi:10.1186/s12974-025-03449-9)
Supplement: Supplementary file 2 — Supplementary Material 2: Supplemental Figure 2. [file 12974_2025_3449_MOESM2_ESM.pdf]

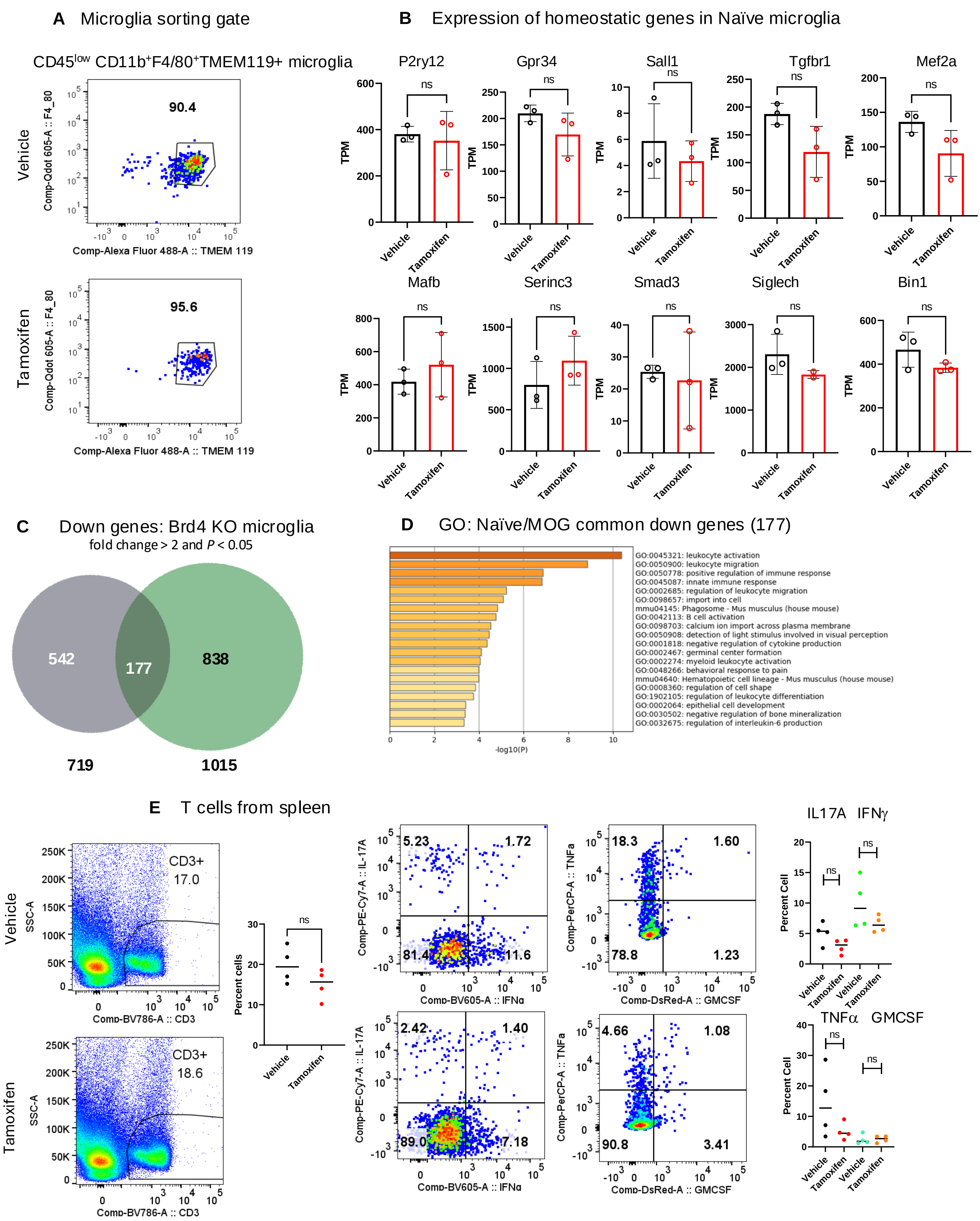

A Microglia sorting gate
CD45low CD11b+F4/80+TMEM119+ microglia
Vehicle
Tamoxifen
90.4
95.6
Comp-Qdot 605-A :: F4_80
Comp-Alexa Fluor 488-A :: TMEM 119
B Expression of homeostatic genes in Naïve microglia
P2ry12
Gpr34
Sall1
Tgfbr1
Mef2a
Mafb
Serinc3
Smad3
Siglech
Bin1
TPM
ns
Vehicle
Tamoxifen
C Down genes: Brd4 KO microglia
fold change > 2 and P < 0.05
542
177
838
719
1015
D GO: Naïve/MOG common down genes (177)
GO:0045321: leukocyte activation
GO:0050900: leukocyte migration
GO:0050778: positive regulation of immune response
GO:0045087: innate immune response
GO:0002685: regulation of leukocyte migration
GO:0098657: import into cell
mmu04145: Phagosome - Mus musculus (house mouse)
GO:0042113: B cell activation
GO:0098703: calcium ion import across plasma membrane
GO:0050908: detection of light stimulus involved in visual perception
GO:0001818: negative regulation of cytokine production
GO:0002467: germinal center formation
GO:0002274: myeloid leukocyte activation
GO:0048266: behavioral response to pain
mmu04640: Hematopoietic cell lineage - Mus musculus (house mouse)
GO:0008360: regulation of cell shape
GO:1902105: regulation of leukocyte differentiation
GO:0002064: epithelial cell development
GO:0030502: negative regulation of bone mineralization
GO:0032675: regulation of interleukin-6 production
-log10(P)
E T cells from spleen
Vehicle
Tamoxifen
CD3+
17.0
18.6
SSC-A
Comp-BV786-A :: CD3
Percent cells
ns
5.23
1.72
81.4
11.6
2.42
1.40
89.0
7.18
Comp-PE-Cy7-A :: IL-17A
Comp-BV605-A :: IFNα
18.3
1.60
78.8
1.23
4.66
1.08
90.8
3.41
Comp-PerCP-A :: TNFa
Comp-DsRed-A :: GMCSF
IL17A IFNγ
TNFα GMCSF
Percent Cell

Supplemental Figure 2 (A) Microglia sorting strategy (B) Quantification of relative TPM values of representative homeostatic genes from Naïve microglia. Significance of differences were determined by unpaired t-test with Welch's correction. (C) Venn diagram comparing down regulated genes from Naïve and MOG immunized microglia. (D) GO analysis of 177 down regulated genes common to naïve and MOG immunized microglia. (E) Flow cytometric analysis of peripheral T cells from spleen of MOG immunized mice. (right) Quantification of T cell percentages expressing IL17a, IFNg, TNFa and GMCSF are not statistically different among vehicle and Tamoxifen treated samples.
