# Supplementary material for: Brd4 expression in CD4 T cells and in microglia promotes neuroinflammation in experimental autoimmune encephalomyelitis
Source: J Neuroinflammation. 2025 Jun 2;22:148. doi: 10.1186/s12974-025-03449-9 (PMC12131476; doi:10.1186/s12974-025-03449-9)
Supplement: Supplementary file 3 — Supplementary Material 3: Table S1. [file 12974_2025_3449_MOESM3_ESM.pdf]

Table S1. Key resources table

REAGENT or RESOURCE	SOURCE	IDENTIFIER
**Antibodies**		
Rabbit anti-Iba1	Fujifilm	Cat# 019-19741, RRID:AB839504
Rat anti-CD3	Abcam	Cat# ab82251, RRID:AB_1658447
Rat anti-mouse I-A/I-E	BioLegend	Cat# 107601, RRID:AB_313316
Goat anti-mouse Alexa Fluor 488	Invitrogen	Cat# A-11001, RRID AB_2534069
Goat anti-rabbit Alexa Fluor633	Invitrogen	Cat# A-21070, RRID AB_2535732
FITC anti-mouse CD4	BioLegend	Cat# 100405, RRID:AB_312690
PE/Cy7 anti-mouse IL17A	BioLegend	Cat# 506922, RRID:AB_2125010
BV605 anti-mouse IFN-g	BioLegend	Cat# 505840, RRID:AB_2734493
Pacific Blue anti-mouse/human CD11b	BioLegend	Cat# 101224, RRID:AB_755986
APC/Cy7 anti-mouse CD45	BioLegend	Cat# 103116, RRID:AB_312981
BV605 anti-mouse F4/80	BioLegend	Cat# 123133, RRID:AB_2562305
Alexa Fluor 488 anti-mouse TMEM119	BioLegend	Cat# 853318
PE/Cy7 anti-mouse CD11c	Biolegend	Cat# 117318, RRID:AB_493568
Alexa Fluor® 700 anti-mouse I-A/I-E	Biolegend	Cat# 107622, RRID:AB_493727
PE/Cy5 CD80	BioLegend	Cat# 104712, RRID:AB_313133
**Chemicals**		
Tamoxifen	Sigma	Cat# T5648-1G
Corn oil	Sigma	Cat# C8267-500ml
Sucrose	Sigma	Cat# 84097-5KG
Paraformaldehyde	Electron Microscopy Sciences	Cat#15714
Hoechst 33342	ThermoFisher	Cat# H1399
Trizol reagent	Invitrogen	Cat#15596026
Percoll	Cytiva	Cat#17089102
Luxol Fast Blue	Abcam	Cat#ab150675
Hematoxylin & Eosin	Abcam	Cat#ab245880
Goat Serum	Gibco	Cat#16210-064
Triton X-100	Sigma	Cat# X100-100ml
**Experimental models: organisms/strains**		
C57BL/6 Brd4 $^{Fl/Fl}$ mice		EMBO Journal (2019) 38: e100293
B6.Cg-Tg*(Cd4-cre)1Cwi/BfluJ*	The Jackson Laboratory	Strain #:022071 RRID:IMSR_JAX:022071
B6.129P2(C)-*Cx3cr1*$^{tm2.1(cre/ERT2)Jung}$/J	The Jackson Laboratory	Strain #:020940
