# Supplementary material for: Brd4 expression in CD4 T cells and in microglia promotes neuroinflammation in experimental autoimmune encephalomyelitis
Source: J Neuroinflammation. 2025 Jun 2;22:148. doi: 10.1186/s12974-025-03449-9 (PMC12131476; doi:10.1186/s12974-025-03449-9)

Name: In vitro T cell differentiation

Additional File 1

Description: Naïve T cells from Brd4$^{+/+}$ CD4 Cre and Brd4$^{f/f}$ CD4 were cultured in different skewing condition for 5 days. Results show that Brd4 is necessary for T cell differentiation in vitro (left). On the right CD45.2 and CD45.1 cells were mixed 1:1. Cells were cultured similarly as above in the presence of CFSE. Result indicated Brd4 supports proliferation of T cells in differentiated condition in vitro.

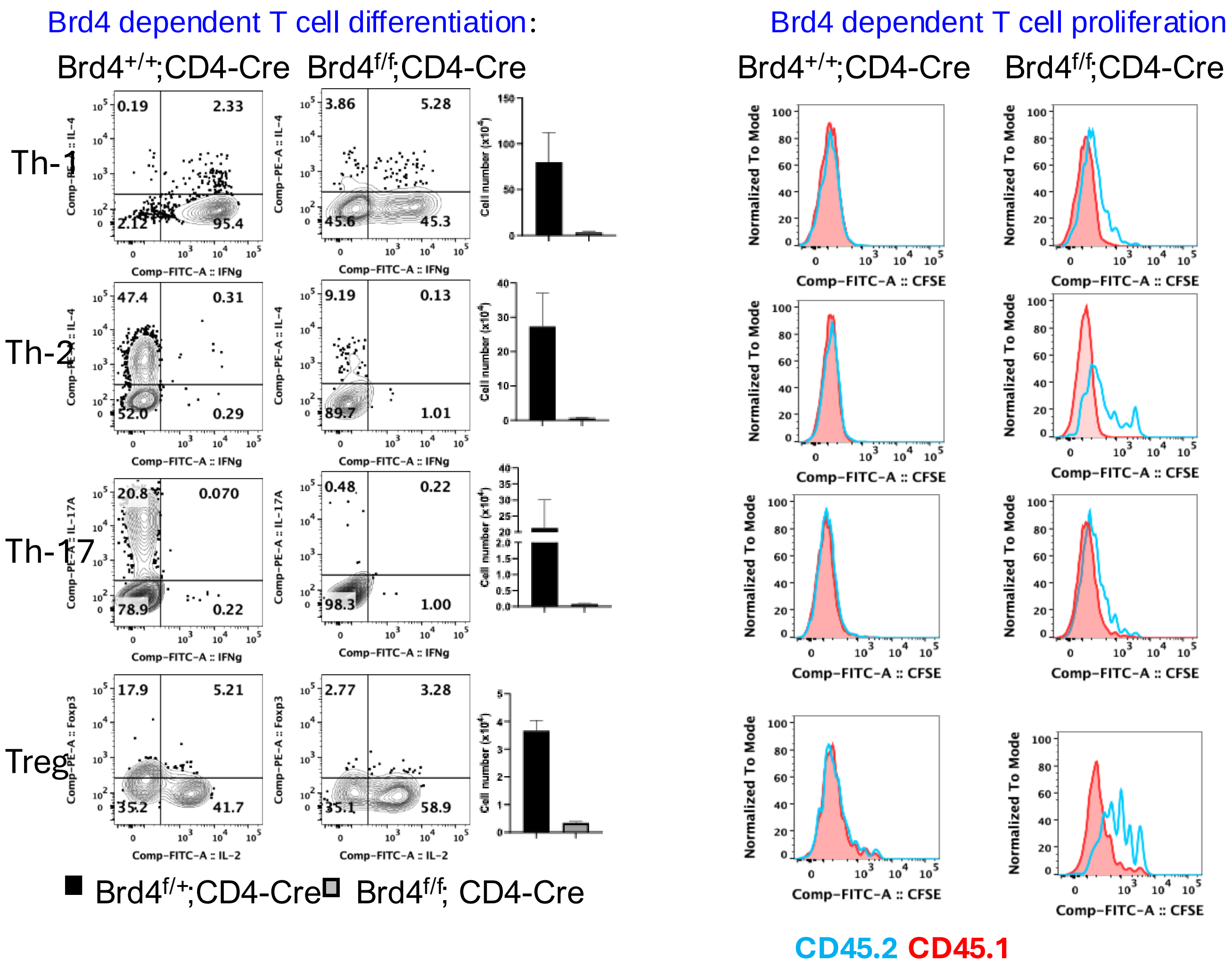

Supplement: Supplementary file 4 — Additional file 1. [file 12974_2025_3449_MOESM4_ESM.pdf]
