# Supplementary material for: Brd4 expression in CD4 T cells and in microglia promotes neuroinflammation in experimental autoimmune encephalomyelitis
Source: J Neuroinflammation. 2025 Jun 2;22:148. doi: 10.1186/s12974-025-03449-9 (PMC12131476; doi:10.1186/s12974-025-03449-9)

Additional File 2

Name: BBB integrity assay

Description: To determine BBB integrity, Evan's blue was injected intravenously.
After an hour mice were perfused incardially. Extravasated Evan's blue was TCA extracted, and quantified by fluorometry. Extravasation was profuse but similar among immunized brains. Naïve brains showed no extravasation. LPS treated samples were used as positive control

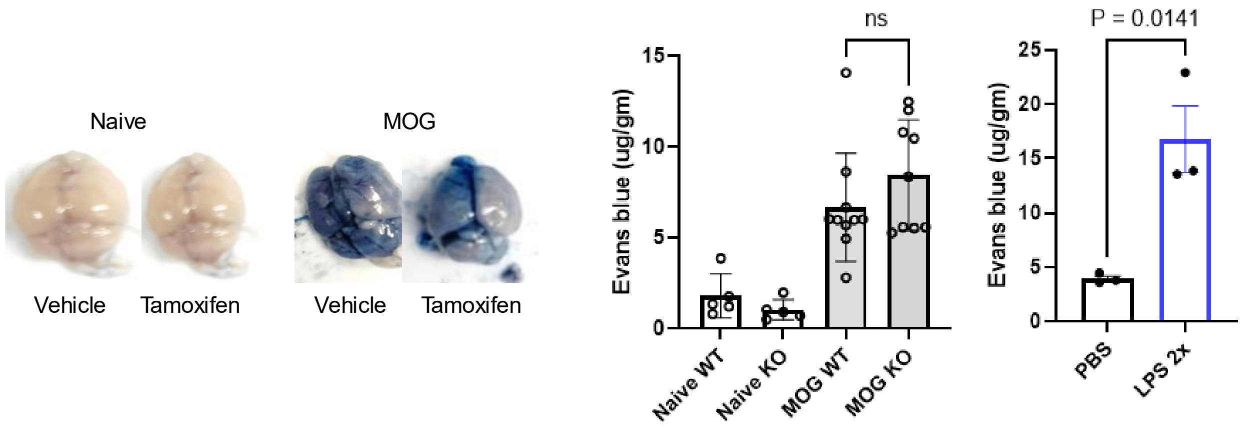

Supplement: Supplementary file 5 — Additional file 2. [file 12974_2025_3449_MOESM5_ESM.pdf]
