# Supplementary material for: Brd4 expression in CD4 T cells and in microglia promotes neuroinflammation in experimental autoimmune encephalomyelitis
Source: J Neuroinflammation. 2025 Jun 2;22:148. doi: 10.1186/s12974-025-03449-9 (PMC12131476; doi:10.1186/s12974-025-03449-9)

Name: EAE Clinical scores to test Tamoxifen effect on EAE symptoms

Description: Brd4$^{f/f}$ or Brd4$^{f/f}$;Cx3cr1 mice were Tamoxifen treated (in red), immunized and subsequently clinical scores were recorded. Course of pathogenesis of Tamoxifen treated Brd4$^{f/f}$ and vehicle injected Brd4$^{f/f}$;Cx3cr1 (black) (Fig. 2C) were similar

Additional File 3

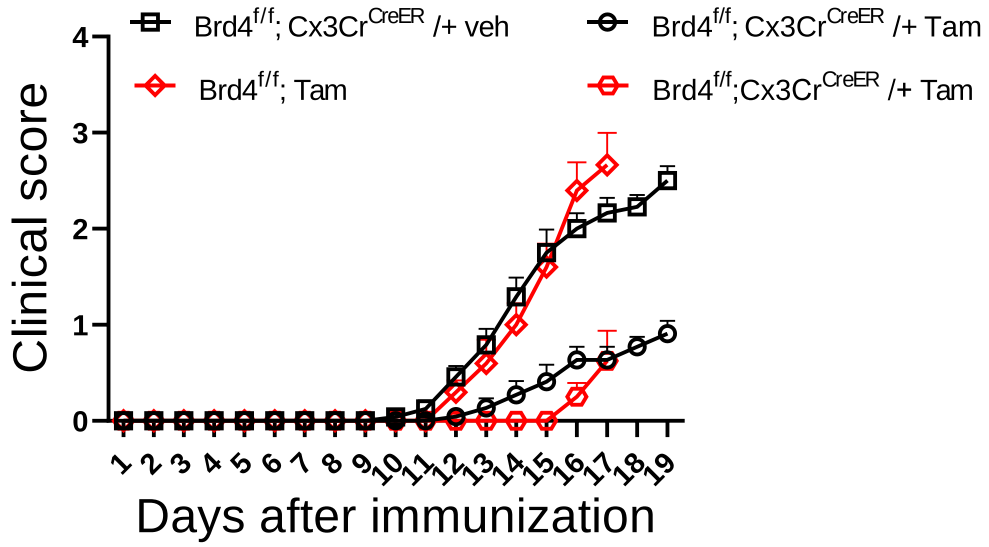

Supplement: Supplementary file 6 — Additional file 3. [file 12974_2025_3449_MOESM6_ESM.pdf]
