# Supplementary material for: Brd4 expression in CD4 T cells and in microglia promotes neuroinflammation in experimental autoimmune encephalomyelitis
Source: J Neuroinflammation. 2025 Jun 2;22:148. doi: 10.1186/s12974-025-03449-9 (PMC12131476; doi:10.1186/s12974-025-03449-9)

Additional File 4

Name: Histological section from spinal cord stained with Luxol fast blue and H&E
Description: Luxol blue was used to assess demyelination across the length of spinal cord. Two images are from thoracic and one from lumber region from each mouse. H&E staining shows influx of invading cells. Demyelination and cell invasion occurred only in vehicle injected Immunized mice.

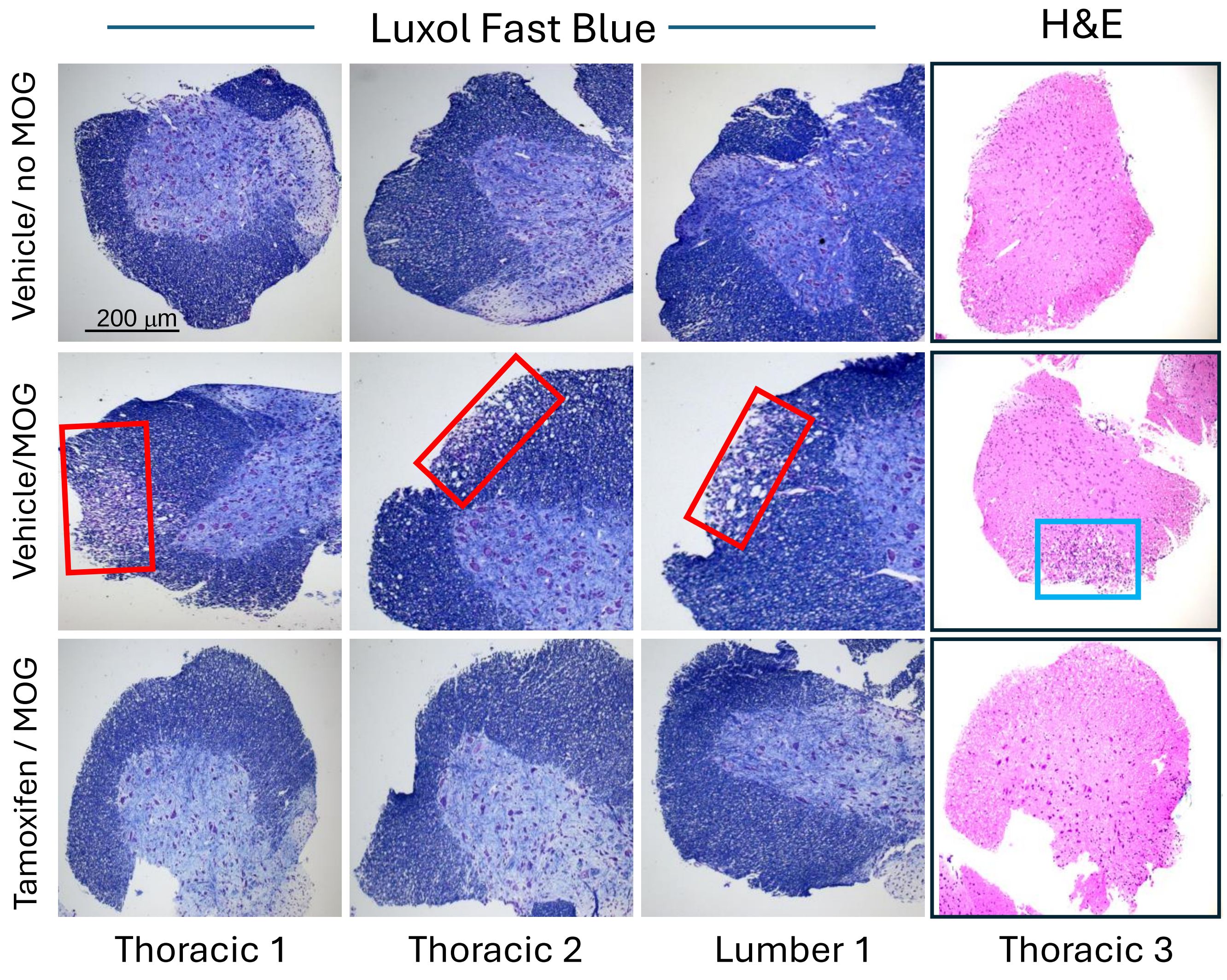

Supplement: Supplementary file 7 — Additional file 4. [file 12974_2025_3449_MOESM7_ESM.pdf]
