# Supplementary figures and images for: Brd4 expression in CD4 T cells and in microglia promotes neuroinflammation in experimental autoimmune encephalomyelitis
Source: J Neuroinflammation. 2025 Jun 2;22:148. doi: 10.1186/s12974-025-03449-9 (PMC12131476; doi:10.1186/s12974-025-03449-9)

## Go analysis of Up regulated genes from Naïve microglia

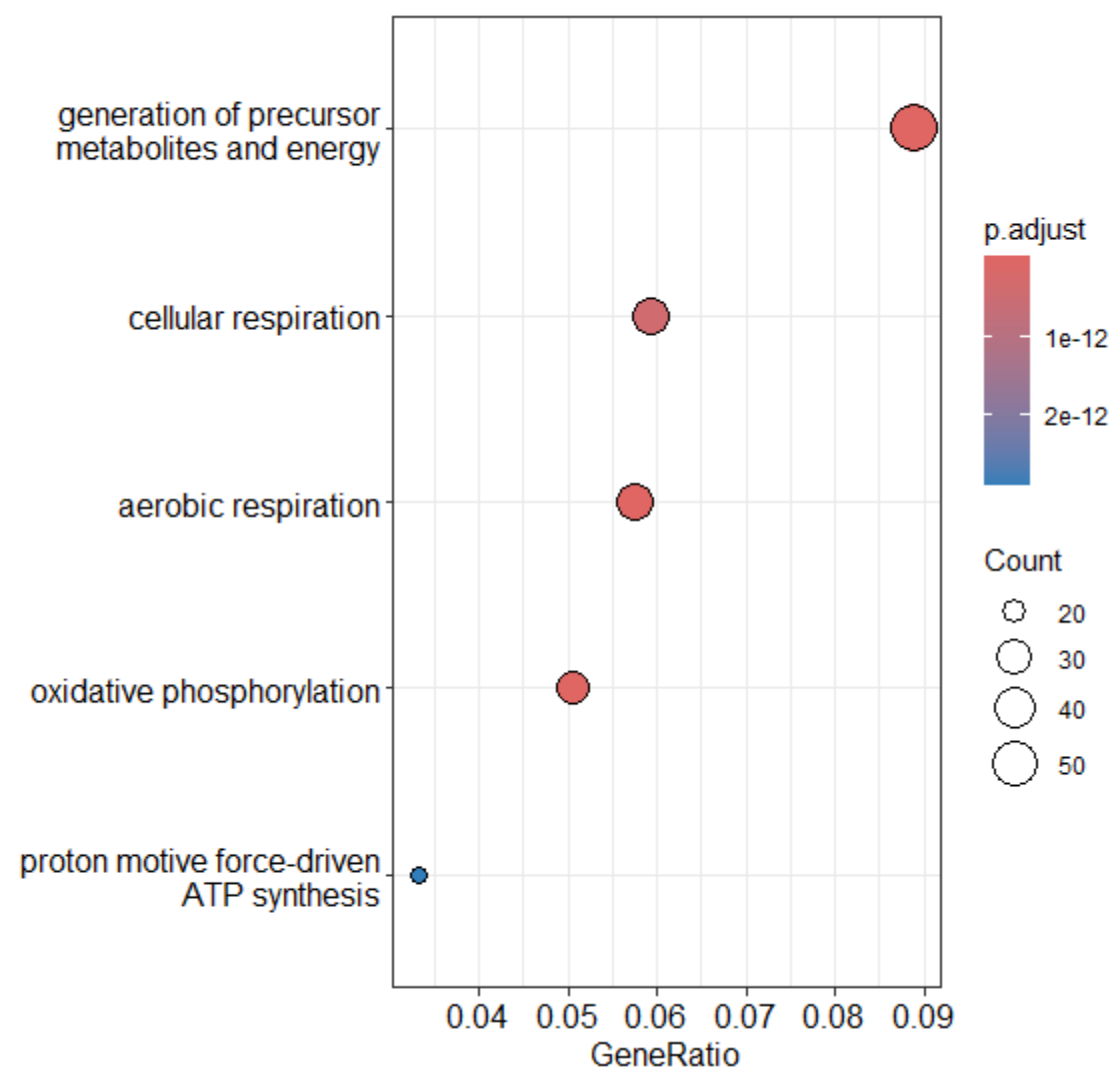

Supplement: Supplementary file 8 — Additional file 5. [file 12974_2025_3449_MOESM8_ESM.pdf]
